# Halo Measurements in Simulated Multifocal Intraocular Lenses

**DOI:** 10.1167/tvst.15.5.21

**Published:** 2026-05-22

**Authors:** Petros Papadogiannis, Victor Rodriguez-Lopez, Alejandra Varea, Diego Chorrero, Lucie Sawides, Carlos Dorronsoro, Alberto de Castro

**Affiliations:** 1 Instituto de Óptica “Daza de Valdés,” Consejo Superior de Investigaciones Científicas, Madrid, Spain; 2 2EyesVision SL, Madrid, Spain

**Keywords:** multifocal IOL simulations, halo assessment, psychophysical testing, preoperative testing, halo perception

## Abstract

**Purpose:**

To quantify the halo size produced by simulated multifocal and extended depth of focus (EDOF) intraocular lenses (IOLs) using a novel psychophysical method, and to compare the results with an established halo assessment tool, computational simulations, and subjective bothersomeness scores.

**Methods:**

Ten participants were each presented with ten IOL designs (three trifocals, three EDOFs, three bifocals, and one monofocal) simulated using SimVis Gekko visual simulator (2EyesVision, Spain). Halo size was quantified using both the established Halo V1.0 software and a custom-developed scrolling method designed for rapid halo assessment. Results were compared with computational predictions derived from Fourier optics. Perceived visual disturbance was evaluated using a shortened version of the Assessment of Intra Ocular Lens Implant Symptoms questionnaire.

**Results:**

Trifocals produced larger halos than EDOFs, whereas the monofocal generated the smallest. Measurements obtained with Halo V1.0 and scrolling method were highly correlated (*r* = 0.99) and showed strong agreement with computational estimations (*r* > 0.92). Halo size increased with IOL addition power by 5 arcmin/diopter. The scrolling method required less time (29.6 ± 16.2 vs. 283 ± 1.7 seconds) and exhibited lower variability (1 ± 0.8 vs. 5.4 ± 5.8 arcmin) compared to Halo V1.0. Subjective disturbance scores correlated strongly with measured halo (*r* > 0.98).

**Conclusions:**

SimVis Gekko emulates halo phenomena associated with different IOL designs. The custom scrolling method provides fast, reliable halo assessment with potential for research and clinical application.

**Translational Relevance:**

Findings support integrating preoperative halo simulation and rapid psychophysical evaluation to enhance personalized IOL selection and patient counseling.

## Introduction

Despite the success of premium intraocular lenses to restore vision at different distances in presbyopic subjects,^1^ these corrections are often associated with dysphotopsias. These unwanted visual phenomena are one of the most common reason for patient dissatisfaction and are often divided for its study into positive and negative type.^2^^,^^3^ Positive dysphotopsias include different kinds of glare such as halos (rings of light), starburst (radiating pattern of light spikes), streaks of light (bright straight lines), and snowballs (cloudy circular spots).^4^ Positive dysphotopsias can occur because of reflections at the edge of the intraocular lens (IOL) or internal reflections on the lens surfaces, and they can also arise from the optics of the IOL itself, designed to provide a particular depth of focus. This optical design can produce the simultaneous perception of more than one image, with a focused image superimposed on one or more out-of-focus images.^5^ Negative dysphotopsias have been attributed to factors such as the distance between the iris and the IOL, the relationship between the anterior capsule and IOL optics, and certain surgical techniques.^2^

The impact of dysphotopsias on the quality of life of patients is significant and can lead to dissatisfaction after lens surgery. The prevalence of bothersomeness because of positive dysphotopsias in the early period after crystalline lens replacement surgery is 50%.^6^ The phenomenon often resolves spontaneously, but in up to 2.2% of cases, symptoms persist.^6^ Negative dysphotopsias are less common and affects approximately 15% of patients in the early postoperative stage, with the symptoms resolving over time in most cases.^7^ Multifocal IOLs are linked to a higher prevalence of positive dysphotopsias compared to monofocals,^8^^,^^9^ with diffractive IOL being more sensitive to glare phenomena than refractive designs.^10^ Halo brightness and size are directly related to light energy distribution and the addition power, with higher addition producing larger halos.^5^

Dissatisfaction after IOL implantation remains an inherent risk, since, before surgery, the only tools available to inform patients are patients’ testimonials and marketing materials. However, the halo experience can largely differ between patients because of the interaction of the IOL with their individual ocular optics and higher-order aberrations.^11^^,^^12^ The most common method to evaluate the presence of halos is the Food and Drug Administration–approved AIOLIS questionnaire—Assessment of Intra Ocular Lens Implant Symptoms—^13^ which assesses the frequency and severity of visual symptoms such as halos, snowballs, streaks, or hazy vision. Several instruments have been developed for a quantitative assessment of the halo size or the associated visual disturbance.^14^^–^^21^ Among them, Halo V1.0 software^19^ is used in the literature because of its ease of deployment across devices, although it is not a validated gold standard and presents constraints related to the psychophysical task. Overall, these tools have limited clinical relevance primarily by the absence of a gold-standard method, a standardized metric, and the strong dependence of measured halo size on pupil size and stimulus parameters, which reduces the repeatability and comparability across studies.^15^^,^^17^^,^^22^ This dual variability—individual optics and methodological—limits the prognostic value of existing measures and highlights the need for tools that allow patients to evaluate their perceived visual quality with different IOL designs.

SimVis Gekko (2EyesVision, Madrid, Spain) is a see-through wearable visual simulator that allows patients to experience the real world through visual simulations of multifocal IOL corrections before IOL implantation.^23^^–^^25^ This binocular device uses programmable tunable lenses operating under the principle of temporal multiplexing,^26^ which modulate optical power at speeds exceeding the flicker-fusion frequency of the human visual system and thereby generating a realistic simulation of the retinal image with multifocal corrections.^27^ In this study, SimVis Gekko was used to simulate vision with different multifocal and extended depth of focus (EDOF) corrections and with a monofocal IOL. A rapid psychophysical method to subjectively quantify the size of the halo was proposed and compared against the results obtained with Halo V1.0 software.^19^ The results were also compared with estimates from computer simulations, and with the responses to a shortened version of the AIOLIS questionnaire.^13^ Together, these analyses evaluate the feasibility and agreement of a preoperative, patient-specific approach to halo evaluation.

## Methods

### Setup

A white LED of the same type was used as a halo source in both psychophysical experiments (Scrolling and Halo V1.0, see below). The diameter of the LED was 1.2 mm (1.03 arcmin), and the emission was 0.094 cd. Psychophysical measurements were performed on a flat screen, and the LED was carefully placed in its center. The screen was a 43ʺ LG (43UR78006LK, UHD 4K) with a size of 95.2 × 53.5 cm, a spatial resolution of 3840 × 2160 pixels. The maximum emission of the display was 196.45 cd/m^2^. The subject was located at a distance of 4 m from the screen, wearing the SimVis Gekko visual simulator (2EyesVision)^28^ that was programmed to simulate different corrections. Each simulated IOL condition was implemented as a time-multiplexed sequence of discrete optical powers delivered by the tunable lens. The induced optical powers and their temporal duty cycle were calculated to emulate the far and near (and, when applicable, intermediate) foci of the corresponding IOL design, which was characterized using a high-speed focimeter.^30^^,^^31^ All simulations were performed with an effective system aperture corresponding to a 3 mm entrance pupil. In this study, intersubject differences in natural pupil diameter under the test conditions are not expected to affect either the SimVis simulations or the halo size estimates because a SimVis Gekko aperture of 3 mm defines the effective aperture of the system.

### Simulated Lenses

Three commercial trifocal lenses (Synergy, PanOptix, FineVision), three commercial EDOF lenses (Vivity, Symfony, Isopure), three generic bifocals with additions of 1.50, 3.00, and 4.50 D, and a monofocal IOL were simulated with SimVis Gekko. The Table presents the specifications (manufacturer, type, optical technology, addition) of the IOLs under study. The subject was kept unaware of the correction presented and the order of the corrections was randomized for each subject. The maximum addition listed in the Table is the dioptric distance for near vision for the trifocal corrections, and the depth of focus for the EDOFs as reported by Rampat and Gatinel.^31^ Three different experiments were conducted sequentially with each correction.

**Table.** Specifications of the Simulated IOLs

Lens	Manufacturer	Type	Optical Technology	Maximum Addition
Synergy	Johnson & Johnson	Trifocal	Diffractive	2.55
PanOptix	Alcon	Trifocal	Diffractive	2.35
FineVision	BVI	Trifocal	Diffractive	2.70
Vivity	Alcon	EDOF	Refractive	1.53
Symfony	Johnson & Johnson	EDOF	Diffractive	1.50
Isopure	PhysIOL	EDOF	Refractive	1.00
Generic Bifocal 3	—	Bifocal	—	4.50
Generic Bifocal 2	—	Bifocal	—	3.00
Generic Bifocal 1	—	Bifocal	—	1.50
Monofocal	—	Monofocal	—	0.00

### Experiment 1. Scrolling Method

A custom-developed method (Scrolling method) presented four 6-arcmin peripheral dots simultaneously, one along each cardinal semi-meridian (0°, 90°, 180°, and 270°). The subject was asked to move the position of the dots by scrolling the mouse wheel to determine the size of the perceived halo. Each mouse wheel-tick displaced all four dots radially 0.025° (i.e., 7 pixels) closer or further away from the led. Two different versions of this method were used: (1) Out-In, with the dots initially visible at 2.5° outside the center so that the subject moved them inward until each dot came in contact with the perceived halo boundary produced by the central LED; and (2) In-Out, with the dots initially occluded by the central LED so that subjects moved them outward until each fully emerged from the halo region. Each version (Out-In, In-Out) comprised three repetitions (six repetitions in total per condition), and the final position of the dots was recorded. The average between Out-In and In-Out results was calculated, and the correlation between both was studied. Measurement time was recorded. Both methods were implemented in MATLAB (R2021b; MathWorks, Natick, MA, USA) with Psychtoolbox (v3.0),^32^ which controlled dots presentation and recorded subjects’ responses. An auditory cue was provided to signal the onset of each stimulus presentation.

### Experiment 2. Halo V1.0

The open-source Halo Software V1.0 (Halo V1.0; University of Granada, Granada, Spain)^19^ was used to randomly present a light gray 5.4 arcmin-diameter dot at different eccentricities from the center along the four cardinal meridians (0°, 90°, 180°, 270°). The task of the observer was to perform a single-interval yes/no detection and to indicate with a left-click whether the dot was visible. The dots were sequentially displayed at 10 different equispaced positions per meridian, ranging between 0.08 and 1.08 degrees from the center of the screen. The dots were presented in each position three times for 1.0 second, and the inter-stimulus interval randomly varied between 1.0 and 1.5 seconds. Measurement time was recorded with a stopwatch.

### Experiment 3. AIOLIS Questionnaire

**Figure 1.**
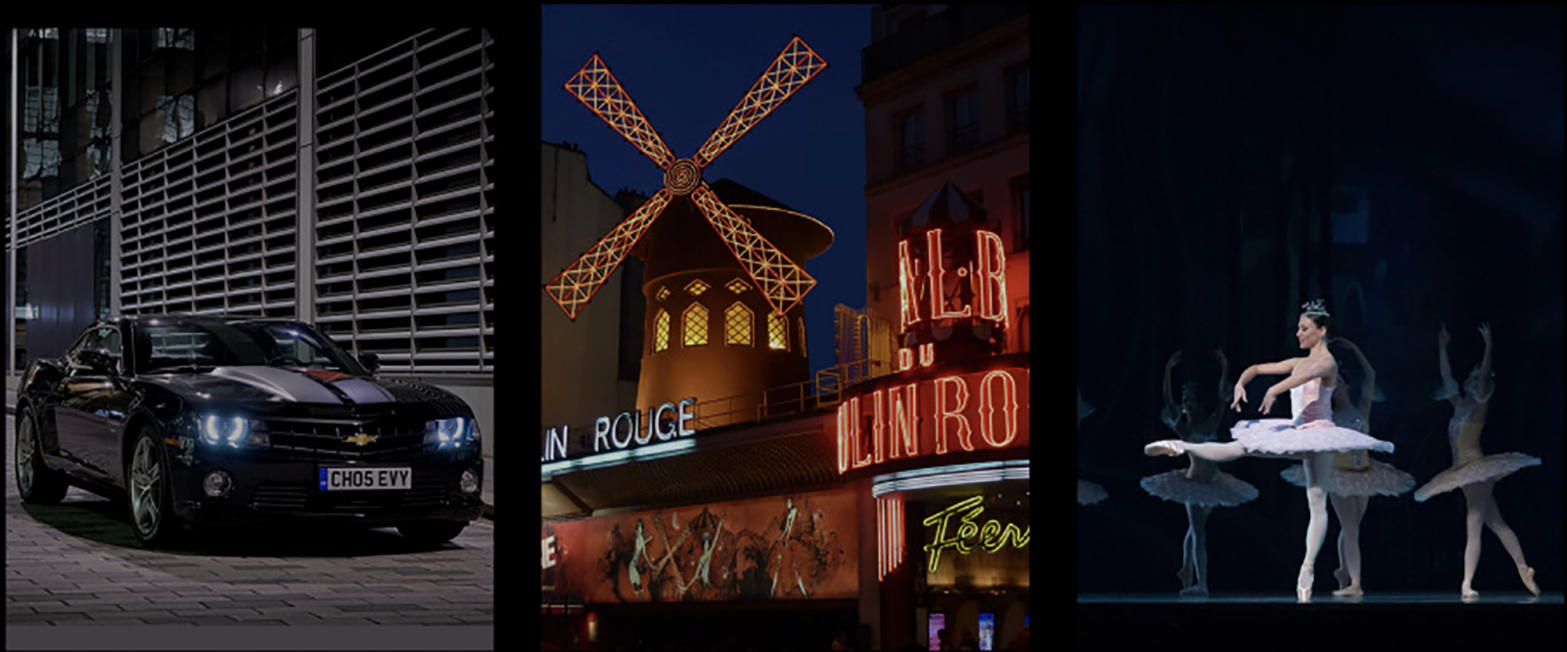
Images presented on the screen to evaluate photic phenomena. Two LEDs were placed in front of the headlights of the car to introduce halos and to mimic night-driving conditions.

To evaluate the visual symptoms produced by the corrections, the subjects answered a shortened version of the AIOLIS questionnaire^13^ while SimVis Gekko simulated each IOL. The participants were presented with three distant urban night scenes: a car, a street scene with neon lights, and a ballet show (see Fig. 1). Two LEDs (same size and intensity as the LED used in the previous measurements) were positioned in front of the car headlights to introduce halos and to mimic night-driving conditions. The images and the LEDs to introduce halos have been used in previous studies.^33^^,^^34^ After showing explanatory images from the AIOLIS questionnaire to define glare, rings, spider webs, and hazy vision, the following three questions were asked for each simulated IOL: (Q1) “How much are you bothered by the glare?”; (Q2) “How much are you bothered by the rings and spider webs?”; and (Q3) “How much are you bothered by the hazy vision?” The order of IOL simulations was randomized for each subject. Subjects provided one score per question for each simulated IOL (i.e., scores were not collected separately for each image). The subjects responded on a scale from 1 to 5, with 1 = Not at all; 2 = A little; 3 = Somewhat; 4 = Quite a bit; and 5 = Extremely.

### Computer Simulations

The image projected on the retina was simulated to measure the halo size computationally and compare it with the experimental measurements. A flat aperture with a diameter of 1.03 arcmin that simulated the LED was convolved with the point spread function of each multifocal correction calculated using the temporal coefficients.^26^^,^^35^ The resulting image was binarized with an energy threshold of 1% and morphological operators were used to determine the halo size. The optical computations were performed assuming a 3 mm entrance pupil diameter at the IOL plane.

### Subjects

Ten subjects participated in the experiment with the custom-developed software (average age 29.4 ± 7.5 years, range 21 to 41). Ten subjects participated in the experiment with Halo V1.0 software and the AIOLIS questionnaire (average age 33.1 ± 7.6 years, range 27 to 51). Four subjects participated in both experiments. All subjects had best-corrected visual acuity of 0.0 logMAR or better and normal ocular health. Subjects performed the experiments wearing their usual ophthalmic prescription mounted in front of the SimVis Gekko device using custom trial-lens holders. All measurements were conducted binocularly. All procedures conformed to the Declaration of Helsinki and were approved by the CSIC Research Ethics Committee. Written informed consent was obtained from all participants before enrollment. Participants were recruited at the Instituto de Óptica, Madrid, Spain.

### Data Analysis

With the custom-developed method, the halo size was calculated as the average eccentricity of the final stimulus position across the three repetitions. Repeatability was estimated as the standard deviation across repetitions. To compare In-Out vs. Out-In versions, the Pearson correlation coefficient and a least-squares linear model fitting (slope, intercept, and 95% confidence intervals) were calculated.

**Figure 2.**
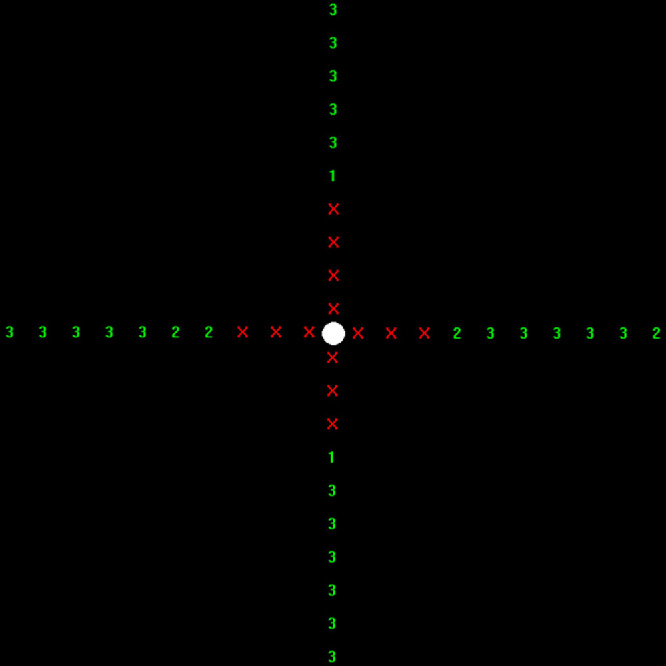
Example of a result from Halo V1.0 software.

Halo V1.0 software results are a detection map with the number of times the dots have been detected by the participant (see Fig. 2). The halo size at each semi-meridian was defined as the first eccentricity with zero detections (red crosses in the example of Fig. 2), and the halo radius was computed as the average across the four semi-meridians. Repeatability was quantified as the standard deviation of the size in repeated measurements.

Normality was assessed with the Shapiro-Wilk test. Differences in halo size across IOL models and IOL groups (trifocals, EDOFs, and bifocals) were evaluated with the Friedman test. To compare the halo size obtained with different measurement methods (Scrolling vs. Halo V1.0 software) a Linear Mixed-Effects model was used. Pairwise comparisons between subjective (Scrolling, Halo V1.0 software) and objective (computational estimation) halo measurements were assessed with Wilcoxon signed-rank tests. Spearman correlation tests were conducted to evaluate the correlation between (i) the three halo-estimation methods, (ii) the halo size and addition power for commercial lenses (as reported in reference 31) and the bifocals, and iii) halo size and responses from the AIOLIS questionnaire. A *P*-value < 0.05 was considered indicative of statistically significant differences.

## Results

The Out-In and In-Out scrolling versions were strongly correlated (Pearson *r* = 0.95, *P* < 0.05). Repeatability, quantified as the across-repetitions standard deviation, was 0.77 arcmin. Based on this result, subsequent analyses report the Scrolling method outcome as the average across Out-In and In-Out estimates.

**Figure 3.**
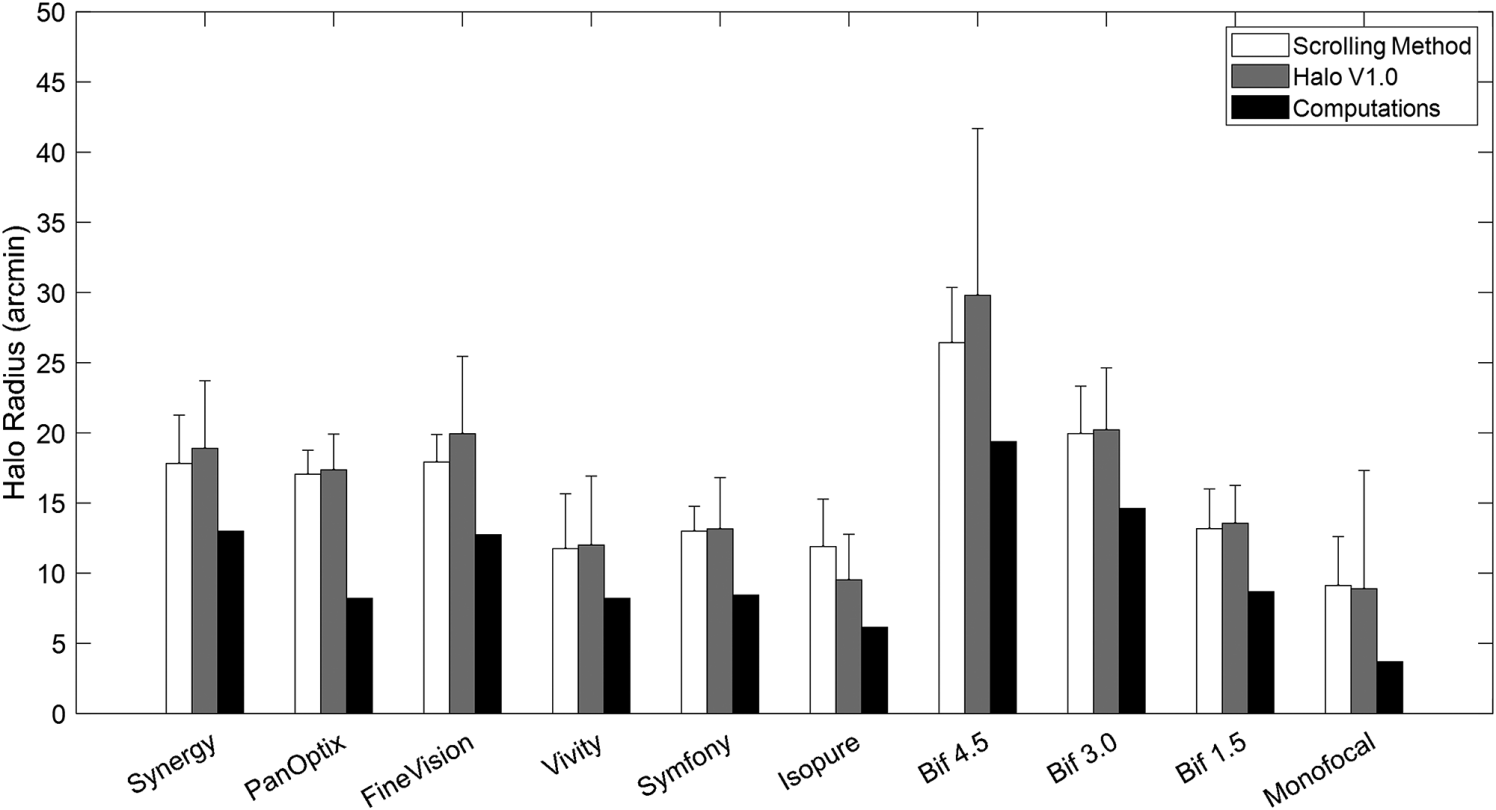
*White*, *gray*, and *black bars* represent the average halo radius across subjects for the Scrolling method, for the Halo V1.0, and for the computations, respectively. The *error bars* represent the standard deviation across subjects.

Figure 3 shows the mean and standard deviation halo radius for each correction obtained with the Scrolling method (the average of Out-In and In-Out estimates), the Halo V1.0 software (first zero-detection location), and the computational estimation. Overall, trifocal lenses produced larger halos compared to the EDOFs, 17.6 ± 2.4 and 12.2 ± 3.1 arcmin on average for the Scrolling method respectively, and 18.7 ± 4.4 and 11.6 ± 4.2 arcmin on average for the Halo V1.0 software. Within groups, FineVision produced the largest halo (17.9 ± 2 arcmin Scrolling method; 19.9 ± 5.5 arcmin Halo V1.0 software) among trifocals and Symfony (13.1 ± 3.7 and 13.0 ± 1.8 arcmin with scrolling and Halo V1.0, respectively) among EDOFs. For the generic bifocals, a higher addition yielded a larger halo size. Across all 10 lenses, the 4.5 D bifocal showed the largest halos (26.4 ± 3.9 arcmin Scrolling method; 29.8 ± 11.9 arcmin Halo V1.0), whereas the monofocal yielded the smallest (9 ± 3 arcmin with both Scrolling and Halo V1.0 methods).

With both subjective methods (Scrolling, Halo V1.0), there were statistically significant differences (Scrolling method: χ^2^(9) = 71.50, *P* < 0.05; Halo V1.0: χ^2^(9) = 71.51, *P* < 0.05) in halo size across IOLs, but no differences within the group of trifocals (Scrolling method: χ^2^(2) = 1.28, *P* > 0.05; Halo V1.0: χ^2^(2) = 3.25, *P* > 0.05). The average halo size obtained with the Scrolling method and the Halo Software V1.0 were not statistically significantly different (*P* > 0.05) and were highly correlated (*r* = 0.99, *P* < 0.05). Pearson correlation between the subjective methods and the computational estimation was *r* = 0.92, *P* < 0.05 for Scrolling-computations, and *r* = 0.94, *P* < 0.05 for Halo V1.0-computations.

**Figure 4.**
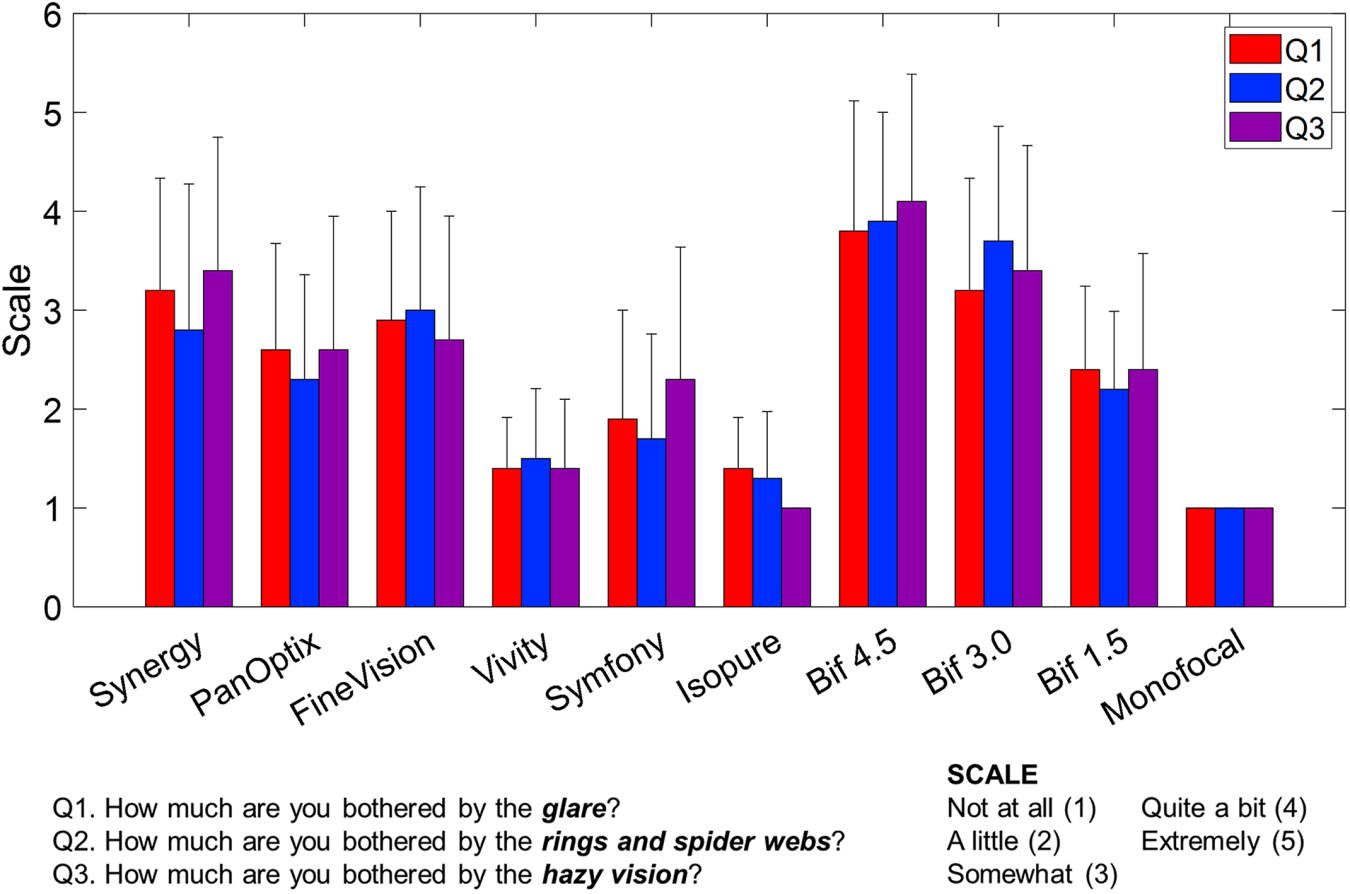
Average and standard deviation of the scores of the AIOLIS questionnaire across subjects that were measured with the Halo V1.0. Each color represents one question.

Figure 4 presents the average scores of the AIOLIS questionnaire where higher scores represent higher disturbances. Halo sizes obtained with Halo V1.0 software were highly correlated with the AIOLIS questionnaire outcomes (*r* > 0.98), indicating that larger halos were associated with greater reported disturbance. On average, the monofocal IOL yielded the lowest score (1 point), the 4.5 D bifocal yielded the highest (3.9 ± 1.2 points), and EDOFs’ average score (1.5 ± 0.9 points) was lower than trifocals average score (2.8 ± 1.2 points). Within-group analyses revealed question-dependent patterns. For Question Q1, no significant differences were observed within trifocals or EDOFs (*P* > 0.05 in both cases), whereas bifocals differed significantly (*P* < 0.05). For Q2, significant differences were found within trifocals and bifocals (*P* < 0.05 in both cases), but not within EDOFs (*P* > 0.05). For Q3, significant differences were found within all groups (trifocals and EDOFs *P* < 0.05, bifocals *P* < 0.001). When AIOLIS responses were averaged across questions, significant within-group effects were present for trifocals (χ^2^(2) = 6.20, *P* < 0.05), EDOFs (χ^2^(2) = 6.74, *P* < 0.05), and bifocals (χ^2^(2) = 19.16, *P* < 0.001). Between-group comparisons showed significant differences for all IOL-group pairs except trifocals vs. bifocals on Q1 (*P* > 0.05) and EDOFs versus Monofocal on Q2 (*P* > 0.05).

**Figure 5.**
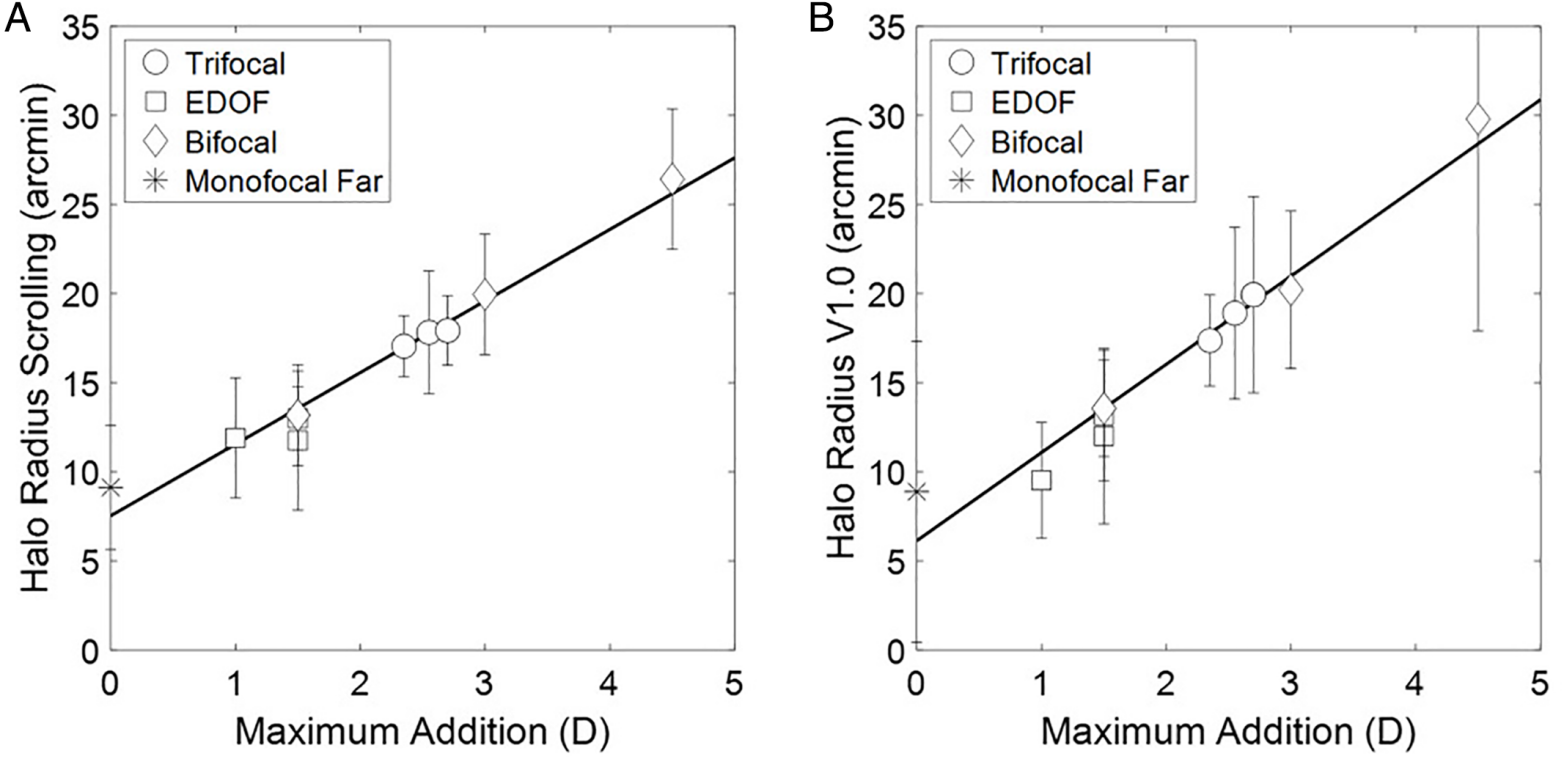
Average and standard deviation across subjects of the halo radius obtained with psychophysical methods versus the addition of the lenses. (**A**) Scrolling. (**B**) Halo V1.0 software.

Figure 5 shows the relationship between the lens maximum addition and halo radius. As described in the Methods section, the maximum addition for the trifocal corrections was the near focus and for the EDOF lenses was the depth of focus. A strong correlation was found for both methods (*r* = 0.98, *P* < 0.05). Linear regression yielded slopes and y-intercepts of 4.02 arcmin/D and 7.54 arcmin and 4.95 arcmin/D and 6.14 arcmin, for Scrolling and Halo V1.0 measurement methods, respectively. These results evidence that the halo radius increases, on average, 4.5 arcmin per diopter of addition.

**Figure 6.**
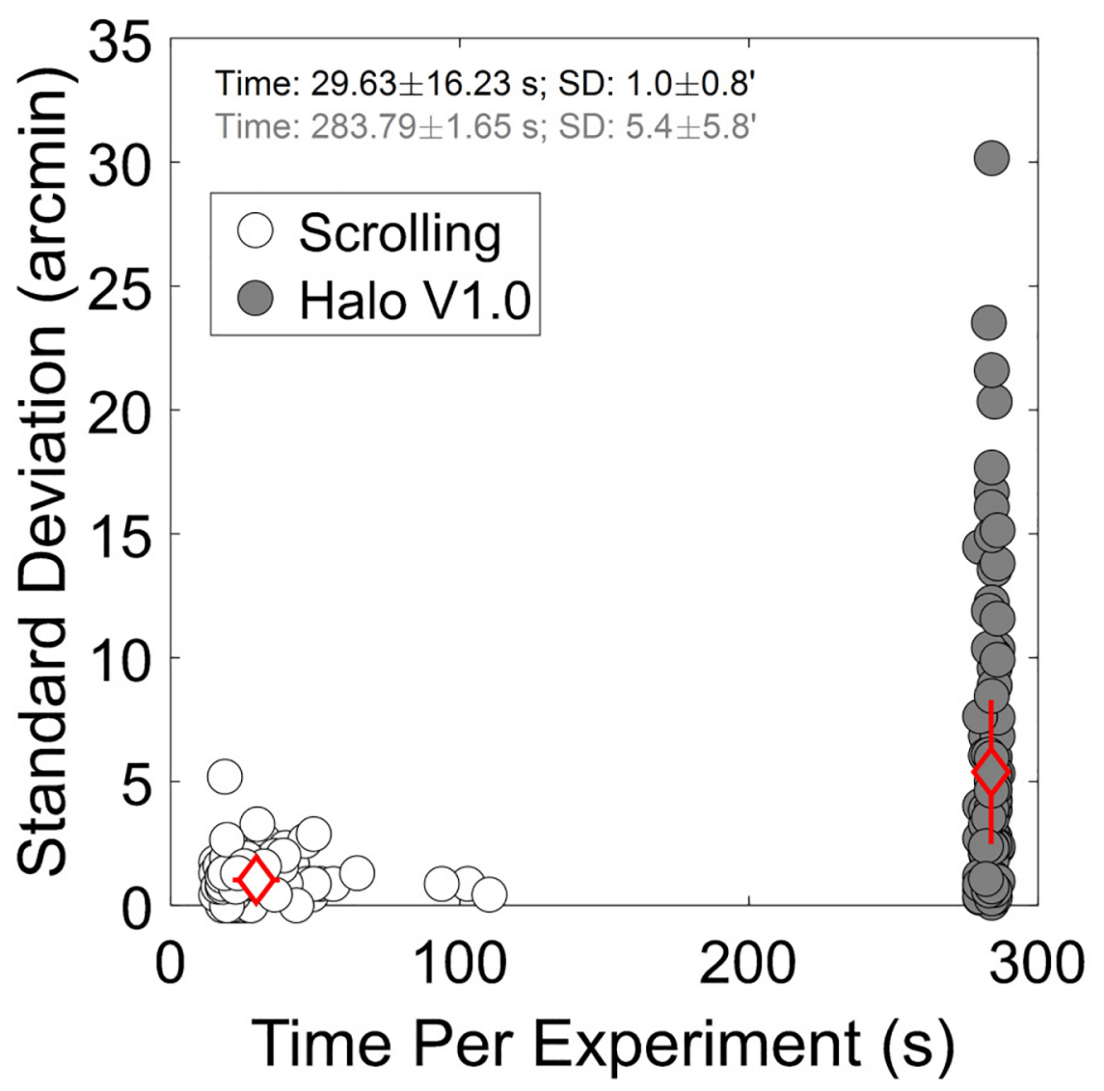
Standard deviation of the determination of the size of the halo and experiment time for the two methods explored. Each dot corresponds to one subject and one condition (*n* = 100). The *red diamonds* represent the average and standard deviation across subjects and conditions.

Figure 6 shows the standard deviation of each method against measurement time per condition for all corrections and subjects and the average and standard deviation value per group (red diamond). On average, the Scrolling method exhibited lower variability than Halo V1.0 software (1 ± 0.8 arcmin and 5.4 ± 5.8 arcmin, respectively). In terms of duration per condition, the Scrolling method was substantially faster than Halo V1.0 (29.6 ± 16.2 and 283.8 ± 1.7 seconds, respectively).

## Discussion

### Subjective Halo Size

This study compared halo size measurements across different IOL designs using two psychophysical methods (a novel Scrolling method and Halo V1.0) and computational estimates. Results confirmed that halo size increases with the addition power of the IOL, in agreement with previous literature on multifocal IOLs.^5^^,^^18^^,^^36^^–^^39^ Among the tested corrections, trifocals intraocular lenses produced larger halos than EDOF corrections, consistent with clinical series reporting higher halo incidence with trifocal optics than with EDOF designs.^40^^,^^41^ High-addition bifocals exhibited the largest halo overall, consistent with several comparative clinical versus on-bench reports showing more frequent or larger halos with trifocal or higher-addition designs versus EDOF.^41^^,^^42^ Although peripheral-target halometry has been described previously,^43^ the present work describes a fast and low-burden implementation (Scrolling) and demonstrates its feasibility and agreement when embedded within a preoperative, real-world simulation workflow (SimVis Gekko).

Although halo sizes obtained from Halo V1.0 and the Scrolling method were highly correlated and did not differ significantly in magnitude, the Scrolling method showed a higher repeatability and a substantially shorter measurement time. The constant-stimuli design of the Halo V1.0 software^19^^,^^44^ requires long test durations (∼284 s per condition), which, together with the absence of an audio cue to alert participants to the onset of each trial, may have contributed to reduced attention and increased variability when measuring with this method. In contrast, the Scrolling method, based on a staircase-like adjustment task, required much less time (∼30 s per condition) and with a fivefold reduction in standard deviation. This aligns with reports that classical halometry approaches are reliable^17^ but are longer to perform than rapid ring-adjustment tasks that take around one to two minutes, similar to our Scrolling method.^22^ The broader distribution of time elapsed to complete the experiment with the Scrolling method when compared to the Halo V1.0 can be explained due to the fact that trial timing is largely software-determined in the second (fixed stimulus duration of 1 second and inter-stimulus interval between 1–1.5 seconds) and that the number of presentations is fixed, leaving limited room for time variation across conditions or participants. In contrast, the Scrolling method is self-paced (participants adjust the target until the task is fulfilled). The Scrolling method requires only a mouse, an LED, and a screen, and thus, given its simplicity, may be more practical for implementation in clinical practice.

Psychophysical halo size was strongly correlated with subjective disturbance scores from the AIOLIS questionnaire^13^; bigger halos bothered subjects more. This is consistent with previous work linking larger halometry measurements to worse optical quality or greater symptomatology,^5^^,^^17^^,^^45^^,^^46^ and is also congruent with previous results using the AIOLIS test.^13^^,^^47^ Nevertheless, some studies report discordance between objective halo metrics and subjective bother, probably reflecting other mechanisms involved, like inter-individual tolerance thresholds and neural adaptation.^17^^,^^38^^,^^48^^,^^49^ Our results suggest that halo size is a reliable predictor of perceived disturbance, at least under controlled preoperative simulations.

### Computational Estimations

Both Halo V1.0 and Scrolling methods correlated strongly with the computational estimates, indicating that simulations successfully captured differences between IOL designs. However, the results differed in absolute values, suggesting a systematic bias in the computed (estimated) halo size. This underestimation in the halo size computed with simulated images of the LED could be due to simplified assumptions in the optical model. We observed that the effect of high-order aberrations was minimal but did not include the effects of polychromatic light.^36^^,^^37^^,^^50^^–^^53^ Also residual misalignments would result in a larger halo size, and the computed estimations did not account for the size of the presented stimulus. Calibration of the model to a perceptual criterion (e.g., matching an encircled-energy proportion to the subjective threshold used in the Scrolling task) and incorporation of subject-specific ocular aberrations could reduce this bias.

### Clinical Implications

Measurements were performed in non-surgical subjects through simulated optics. The frequency cycle of the tunable lens is well above the defocus flicker fusion frequency^54^ and differences in terms of accommodation are not anticipated. The main difference between young subjects and those of higher age can be changes in the transparency of the crystalline lens and the potential appearance of cataracts. However, previous studies using SimVis Gekko have performed measurements on subjects with cataracts before and after cataract surgery and found a high correlation between the visual acuity before surgery with the visual simulation provided by SimVis Gekko and the visual acuity after cataract surgery.^55^^,^^56^ In view of these results, differences between age groups are not expected. In addition, SimVis Gekko captures dysphotopsias primarily related to multifocal image formation (e.g., halo/glare magnitude and haze-like degradation), but it does not explicitly reproduce light scatter/straylight which may produce qualitative patterns such as rings or spider webs and depend on additional optical mechanisms and postoperative factors not modeled in the simulator. Nevertheless, previous multiple studies indicate that see-through visual simulators (SimVis Gekko) can reproduce postoperative through-focus performance and comparative trends with good fidelity in both clear-lens and cataract populations.^25^^,^^57^^–^^60^ After surgery, halo magnitude may remain relatively stable whereas perceived bothersomeness often decreases with time because of adaptation, which has been explicitly tracked in early postoperative intervals using halo tasks.^13^^,^^47^^,^^60^ Future work should compare halo sizes obtained preoperatively using SimVis technology with halo sizes measured postoperatively within the same patients (although no significant differences are anticipated) and across different luminance conditions.

Additionally, modifications of the Scrolling method (e.g., in the number of peripheral dots, or in appearance rules depending on the angle) may enable estimating the directionality or anisotropy of the produced halo, similar to other halometry approaches.^17^^,^^20^ Standardize reporting of the luminance of the stimuli and pupil conditions would enable meta-analysis across studies, as advocated by Kohnen and Suryakumar,^38^ and is readily achievable with the methods demonstrated in the study.

## Conclusions

A new method to measure the size of the halos produced with multifocal corrections was presented and tested using a visual simulator. The method based on approaching peripheral stimuli to the halo by scrolling the mouse-wheel was shown to provide fast and repeatable measurements and has the potential for broad clinical adoption. Our findings support the feasibility of combining preoperative simulations and fast psychophysical halometry as a candidate workflow to facilitate and personalize IOL selection and to advise patients about photic phenomena and motivate future clinical studies evaluating this approach against postoperative outcomes.

## References

[1] Zvorničanin J, Zvorničanin E. Premium intraocular lenses: the past, present and future. *J Curr Ophthalmol*. 2018; 30: 287–296. 30555960 10.1016/j.joco.2018.04.003 PMC6276729

[2] Pusnik A, Petrovski G, Lumi X. Dysphotopsias or unwanted visual phenomena after cataract surgery. *Life*. 2022; 13(1): 53. 36676002 10.3390/life13010053 PMC9866410

[3] Shen W, Zhuo B, Cai L, Yang J. Photic phenomena after presbyopia-correcting intraocular lens implantation: incidence, risk factors, prevention, and strategies. *Expert Rev Med Devices*. 2025; 22: 939–948. 40737187 10.1080/17434440.2025.2539262

[4] de Vries NE, Nuijts R. Multifocal intraocular lenses in cataract surgery: literature review of benefits and side effects. *J Cataract Refract Surg*. 2013; 39: 268–278. 23332253 10.1016/j.jcrs.2012.12.002

[5] Alba-Bueno F, Garzón N, Vega F, Poyales F, Millán MS. Patient-perceived and laboratory-measured halos associated with diffractive bifocal and trifocal intraocular lenses. *Curr Eye Res*. 2018; 43: 35–42. 29161162 10.1080/02713683.2017.1379541

[6] Hu J, Sella R, Afshari NA. Dysphotopsia: a multifaceted optic phenomenon. *Curr Opin Ophthalmol*. 2018; 29: 61–68. 29084005 10.1097/ICU.0000000000000447

[7] Bournas P, Drazinos S, Kanellas D, Arvanitis M, Vaikoussis E. Dysphotopsia after cataract surgery: comparison of four different intraocular lenses. *Ophthalmologica*. 2007; 221: 378–383. 17947823 10.1159/000107496

[8] Chiam PJT, Chan JH, Aggarwal RK, Kasaby S. ReSTOR intraocular lens implantation in cataract surgery: quality of vision. *J Cataract Refract Surg*. 2006; 32: 1459–1463. 16931256 10.1016/j.jcrs.2006.04.015

[9] Hofmann T, Zuberbuhler B, Cervino A, Montés-Micó R, Haefliger E. Retinal straylight and complaint scores 18 months after implantation of the AcrySof monofocal and ReSTOR diffractive intraocular lenses. *J Refract Surg*. 2009; 25: 485–492. 19603616 10.3928/1081597X-20090512-02

[10] Pieh S, Weghaupt H, Skorpik C. Contrast sensitivity and glare disability with diffractive and refractive multifocal intraocular lenses. *J Cataract Refract Surg*. 1998; 24: 659–662. 9610449 10.1016/s0886-3350(98)80261-7

[11] Pierson C, Wienold J, Bodart M. Review of factors influencing discomfort glare perception from daylight. *Leukos*. 2018; 14: 111–148.

[12] Navarro R . The optical design of the human eye: a critical review. *J Optom*. 2009; 2: 3–18.

[13] Hays RD, MacRae S, Holladay J, et al. Development of a patient-reported outcome measure to assess symptoms associated with cataract surgery and intraocular lens implants. *Ophthalmology*. 2023; 130: 715–725. 37055289 10.1016/j.ophtha.2023.02.026

[14] Linhares JMM, Neves H, Lopes-Ferreira D, Faria-Ribeiro M, Peixoto-de-Matos SC, Gonzalez-Meijome JM. Radiometric characterization of a novel LED array system for visual assessment. *J Mod Opt*. 2013; 60: 1136–1144.

[15] Puell MC, Pérez-Carrasco MJ, Barrio A, Antona B, Palomo-Alvarez C. Normal values for the size of a halo produced by a glare source. *J Refract Surg*. 2013; 29: 618–622. 24016347 10.3928/1081597X-20130819-03

[16] Adrian J, Hue D, Porte S, Le Brun J. Validation of the driver ecological glare test. *J Safety Res*. 2020; 72: 139–143. 32199556 10.1016/j.jsr.2019.12.007

[17] Buckhurst PJ, Naroo SA, Davies LN, et al. Tablet App halometer for the assessment of dysphotopsia. *J Cataract Refract Surg*. 2015; 41: 2424–2429. 26703492 10.1016/j.jcrs.2015.05.041

[18] Pieh S, Lackner B, Hanselmayer G, et al. Halo size under distance and near conditions in refractive multifocal intraocular lenses. *Br J Ophthalmol*. 2001; 85: 816–821. 11423456 10.1136/bjo.85.7.816 PMC1724058

[19] Jimenez JR, Anera RG, Castro JJ, et al. Halo v1.0 Software. 2008. Available at: http://hdl.handle.net/10481/5478.

[20] Ferreira-Neves H, Macedo-de-Araújo R, Rico-Del-Viejo L, Da-Silva AC, Queirós A, González-Méijome JM. Validation of a method to measure light distortion surrounding a source of glare. *J Biomed Opt*. 2015; 20(7): 75002. 26146877 10.1117/1.JBO.20.7.075002

[21] Pires LVS, Mendes MG, Belmonte PNA, de Abreu R, de Lima Monteiro DW, Lyra JM. Quantitative evaluation of optical dysphotopsias in IOLs. In: *2025 9th International Symposium on Instrumentation Systems, Circuits and Transducers (INSCIT)*. 2025: 1–6.

[22] Jenkins MD, Alarcon A, Faria Ribeiro M, et al. Pre-clinical methods to evaluate photic phenomena in intraocular lenses. *Biomed Opt Express*. 2024; 15: 6989–6998. 39679401 10.1364/BOE.541022 PMC11640559

[23] Radhakrishnan A, Pascual D, Marcos S, Dorronsoro C. Vision with different presbyopia corrections simulated with a portable binocular visual simulator. *PLoS One*. 2019; 14(8): 1–13. 10.1371/journal.pone.0221144 PMC6701771 31430328

[24] Vinas M, Aissati S, Romero M, et al. Pre-operative simulation of post-operative multifocal vision. *Biomed Opt Express*. 2019; 10: 5801–5817. 31799048 10.1364/BOE.10.005801 PMC6865107

[25] Marcos S, Artal P, Atchison DA, et al. Adaptive optics visual simulators: a review of recent optical designs and applications. *Biomed Opt Express*. 2022; 13: 6508–6532. 36589577 10.1364/BOE.473458 PMC9774875

[26] Akondi V, Dorronsoro C, Gambra E, Marcos S. Temporal multiplexing to simulate multifocal intraocular lenses: theoretical considerations. *Biomed Opt Express*. 2017; 8: 3410–3425. 28717577 10.1364/BOE.8.003410 PMC5508838

[27] Rodriguez-Lopez V, Geisler W, Dorronsoro C. Spatiotemporal defocus sensitivity function of the human visual system. *Biomed Opt Express*. 2023; 14: 3654–3670. 37497500 10.1364/BOE.486242 PMC10368063

[28] Dorronsoro C, Radhakrishnan A, Alonso-Sanz JR, et al. Portable simultaneous vision device to simulate multifocal corrections. *Optica*. 2016; 3: 918–924.

[29] Dorronsoro C, Barcala X, Gambra E, et al. Tunable lenses: dynamic characterization and fine-tuned control for high-speed applications. *Opt Express*. 2019; 27: 2085. 30732252 10.1364/OE.27.002085

[30] Papadogiannis P, Gambra E, Łabuz G, et al. Visual simulation of intraocular lenses: from on-bench performance to computational and experimental validations. *Biomed Opt Express*. 2024; 15: 6521. 39553883 10.1364/BOE.538878 PMC11563315

[31] Rampat R, Gatinel D. Multifocal and Extended depth-of-focus intraocular lenses in 2020. *Ophthalmology*. 2021; 128(11): e164–e185. 32980397 10.1016/j.ophtha.2020.09.026

[32] Brainard DH . The Psychophysics Toolbox. *Spat Vis*. 1997; 10: 433–436. 9176952

[33] Barcala X, Vinas M, Ruiz S, et al. Multifocal contact lens vision simulated with a clinical binocular simulator. *Contact Lens Anterior Eye*. 2022;(May): 101716. 35606298 10.1016/j.clae.2022.101716 PMC12077379

[34] Rodriguez-Lopez V, Barcala X, Zaytouny A, Dorronsoro C, Peli E, Marcos S. Monovision Correction Preference and Eye Dominance Measurements. *Transl Vis Sci Technol*. 2023; 12(3): 18. 10.1167/tvst.12.3.18 PMC10043500 36939712

[35] Papadogiannis P, Gambra E, Łabuz G, et al. Visual simulation of intraocular lenses: from on-bench performance to computational and experimental validations. *Biomed Opt Express*. 2024; 15: 6521–6530. 39553883 10.1364/BOE.538878 PMC11563315

[36] Alba-Bueno F, Vega F, Millán MS. Halos and multifocal intraocular lenses: origin and interpretation. *Arch Soc Esp Oftalmol*. 2014; 89: 397–404. 24951327 10.1016/j.oftal.2014.01.002

[37] Vega F, Alba-Bueno F, Millán MS, Varón C, Gil MA, Buil JA. Halo and through-focus performance of four diffractive multifocal intraocular lenses. *Invest Ophthalmol Vis Sci*. 2015; 56: 3967–3975. 26098463 10.1167/iovs.15-16600

[38] Kohnen T, Suryakumar R. Measures of visual disturbance in patients receiving extended depth-of-focus or trifocal intraocular lenses. *J Cataract Refract Surg*. 2021; 47: 245–255. 32818348 10.1097/j.jcrs.0000000000000364

[39] Vega F, Azor JA, Millán S. Halo in extended depth of focus and bifocal intraocular lenses. *Asian J Physics*. 2022; 31: 663–675.

[40] Cochener B . Clinical outcomes of a new extended range of vision intraocular lens: international multicenter concerto study. *J Cataract Refract Surg*. 2016; 42: 1268–1275. 27697244 10.1016/j.jcrs.2016.06.033

[41] Karuppiah P, Varman NVA, Varman A, Balakumar D. Comparison of clinical outcomes of trifocal intraocular lens (AT LISA, Eyecryl SERT trifocal) versus extended depth of focus intraocular lens (Eyhance, Eyecryl SERT EDOF). *Indian J Ophthalmol*. 2022; 70: 2867–2871. 35918933 10.4103/ijo.IJO_2921_21 PMC9672785

[42] Zhong Y, Wang K, Yu X, Liu X, Yao K. Comparison of trifocal or hybrid multifocal-extended depth of focus intraocular lenses: a systematic review and meta-analysis. *Sci Rep*. 2021; 11(1): 6699. 33758333 10.1038/s41598-021-86222-1 PMC7987991

[43] Pieh S . Halo size under distance and near conditions in refractive multifocal intraocular lenses. *Br J Ophthalmol*. 2001; 85: 816–821. 11423456 10.1136/bjo.85.7.816 PMC1724058

[44] Castro JJ, Ortiz C, Pozo AM, Anera RG, Soler M. A visual test based on a freeware software for quantifying and displaying night-vision disturbances: study in subjects after alcohol consumption. *Theor Biol Med Model*. 2014; 11(1): S1. 25079703 10.1186/1742-4682-11-S1-S1 PMC4108868

[45] Yao L, Xu Y, Han T, et al. Relationships between haloes and objective visual quality in healthy eyes. *Transl Vis Sci Technol*. 2020; 9(10): 13. 10.1167/tvst.9.10.13 PMC7488622 32974085

[46] Giers BC, Khoramnia R, Varadi D, et al. Functional results and photic phenomena with new extended-depth-of-focus intraocular Lens. *BMC Ophthalmol*. 2019; 19: 197. 31462225 10.1186/s12886-019-1201-3 PMC6714397

[47] Masket S, Lum F, MacRae S, et al. Symptoms and satisfaction levels associated with intraocular lens implants in the Monofocal and Premium IOL Patient-Reported Outcome Measure Study. *Ophthalmology*. 2023; 130: 726–734. 37061911 10.1016/j.ophtha.2023.02.027

[48] Rudalevicius P, Lekaviciene R, Auffarth GU, Liutkeviciene R, Jasinskas V. Relations between patient personality and patients‘ dissatisfaction after multifocal intraocular lens implantation: clinical study based on the five factor inventory personality evaluation. *Eye*. 2020; 34: 717–724. 31541219 10.1038/s41433-019-0585-x PMC7093406

[49] Grzybowski A, Kanclerz P, Muzyka-Woźniak M. Methods for evaluating quality of life and vision in patients undergoing lens refractive surgery. *Graefes Arch Clin Exp Ophthalmol*. 2019; 257: 1091–1099. 30824995 10.1007/s00417-019-04270-w

[50] Ravikumar S, Bradley A, Thibos LN. Chromatic aberration and polychromatic image quality with diffractive multifocal intraocular lenses. *J Cataract Refract Surg*. 2014; 40: 1192–1204. 24957438 10.1016/j.jcrs.2013.11.035

[51] Millán MS, Vega F, Ríos-López I. Polychromatic image performance of diffractive bifocal intraocular lenses: longitudinal chromatic aberration and energy efficiency. *Invest Ophthalmol Vis Sci*. 2016; 57: 2021–2028. 27100158 10.1167/iovs.15-18861

[52] Salvá L, García S, García-Delpech S, Martínez-Espert A, Montagud-Martínez D, Ferrando V. Comparison of the polychromatic image quality of two refractive-segmented and two diffractive multifocal intraocular lenses. *J Clin Med*. 2023; 12: 4678. 37510792 10.3390/jcm12144678 PMC10380361

[53] Naujokaitis T, Auffarth GU, Khoramnia R, Łabuz G. Complementary system vs conventional trifocal intraocular lens: comparison of optical quality metrics and unwanted light distribution. *J Cataract Refract Surg*. 2023; 49: 84–90. 36325833 10.1097/j.jcrs.0000000000001082 PMC9794130

[54] Rodriguez-Lopez V, Geisler W, Dorronsoro C. Spatiotemporal defocus sensitivity function of the human visual system. *Biomed Opt Express*. 2023; 14(7): 3654. 37497500 10.1364/BOE.486242 PMC10368063

[55] Carrasco-Rojo S, Giacopinelli L, Baoud Ould Haddi I, García-Montero M, Arriola-Villalobos P, Garzón N. Simulated vs. real visual performance of an EDOF intraocular lens using adaptive optics technology (Preprint posted online February 19, 2026). *Graefes Arch Clin Exp Ophthalmol*. doi:10.1007/s00417-026-07121-7. 41711804

[56] Barcala X, Zaytouny A, Rego-Lorca D, et al. Visual simulations of presbyopic corrections through cataract opacification. *J Cataract Refract Surg*. 2023; 49: 34–43. 35971215 10.1097/j.jcrs.0000000000001040 PMC9794132

[57] Vinas M, Aissati S, Gonzalez-Ramos AM, et al. Optical and visual quality with physical and visually simulated presbyopic multifocal contact lenses. *Transl Vis Sci Technol*. 2020; 9(10): 20. 10.1167/tvst.9.10.20 PMC7509762 33005478

[58] Barcala X, Zaytouny A, Rego-Lorca D, et al. Visual simulations of presbyopic corrections through cataract opacification. *J Cataract Refract Surg*. 2023; 49: 34–43. 35971215 10.1097/j.jcrs.0000000000001040 PMC9794132

[59] Esteban-Ibañez E, Montagud-Martínez D, Sawides L, et al. Simulation of daily soft multifocal contact lenses using SimVis Gekko: from in-vitro and computational characterization to clinical validation. *Sci Rep*. 2024; 14(1): 8592. 38615153 10.1038/s41598-024-59178-1 PMC11016090

[60] Bhogal-Bhamra GK, Aujla M, Kolli S, Sheppard AL, Wolffsohn JS. Glare prediction and mechanism of adaptation following implantation of hydrophilic and hydrophobic intraocular lenses. *Front Ophthalmol*. 2024; 4: 1310468. 10.3389/fopht.2024.1310468 PMC11182291 38984113

